# Discrimination of Cellulose I, II, III_I_ and III_II_ Polymorphs in Cellulosic Fibers by NIR Hyperspectral Imaging Supported by XRD and XPS

**DOI:** 10.3390/polym18091047

**Published:** 2026-04-25

**Authors:** Isidora Reyes-González, Isabel Carrillo-Varela, Natacha Rosales Charlín, Pablo Reyes-Contreras, Lucas Romero-Albornoz, Rosario del P. Castillo, Alistair W. T. King, Fabiola Valdebenito, Regis Teixeira Mendonҫa

**Affiliations:** 1 Facultad de Ciencias Forestales, Universidad de Concepción, Concepción 4070386, Chile; ireyes2017@udec.cl (I.R.-G.); nrosales2019@udec.cl (N.R.C.); 2 Centro de Biotecnología, Universidad de Concepción, Concepción 4070386, Chile; preyesc@udec.cl (P.R.-C.); luromero@udec.cl (L.R.-A.); rosariocastillo@udec.cl (R.d.P.C.); 3 Bioforest SpA, Camino a Coronel Km 15, Coronel 4190000, Chile; isabel.carrillo@arauco.com; 4 Facultad de Farmacia, Universidad de Concepción, Concepción 4070386, Chile; 5 VTT Technical Research Centre of Finland Ltd., Tietorie 4e, 02150 Espoo, Finland; alistair.king@vtt.fi; 6 Centro de Energía, Universidad Católica de la Santísima Concepción, Concepción 4090541, Chile; fvaldebenito@ucsc.cl

**Keywords:** cellulose polymorphs, hyperspectral imaging, X-ray diffraction, X-ray photoelectron spectroscopy, non-destructive characterization

## Abstract

Native cellulose I can be converted into crystalline polymorphs II and III_I_, while cellulose II can be further converted into III_II_ through chemical treatments that induce significant structural, physical, and chemical changes. Accurate identification and differentiation of these polymorphs is essential for predicting fiber reactivity and processing behavior, but current methods are time-consuming. This study demonstrates the potential of near-infrared hyperspectral imaging (HSI-NIR) combined with linear discriminant analysis as a rapid, non-destructive tool for polymorph discrimination. Cellulose I, II, III_I_, and III_II_ were produced from bleached kraft pulps of eucalyptus and pine and from cotton linters using NaOH (20% *w*/*v*) and ethylenediamine treatments. HSI-NIR successfully differentiated polymorphs based on spectral signatures in the 1480–1600 nm range, regardless of botanical source. Complementary characterization by XRD confirmed polymorph conversions, showing crystallinity reductions of 10–15% for cellulose I→II and I→III_I_ conversions, with crystallite size decreasing from 7.2 nm (cotton CI) to 3.2–3.4 nm in all CIII_II_ samples. XPS analysis revealed increased C-O surface accessibility in cellulose II and III, with complete disappearance of COOH groups in cellulose III samples. These results establish HSI as a promising screening tool for cellulose polymorph identification (>95% classification accuracy) and provide comprehensive baseline data on structural and chemical transformations that govern fiber reactivity in chemical and enzymatic processes.

## 1. Introduction

Cellulose is a homopolysaccharide abundant in the cell walls of plants. It is found in a relatively pure native state in cotton fibers. The polymeric structure of cellulose is based on linear chains of β-1,4-linked glucose residues, with a typical degree of polymerization ranging from 50 to 15,000 units, depending on the origin and treatments the cellulose has undergone [1,2]. At the supramolecular level, the structure of cellulose has crystalline and ‘amorphous’ regions. The crystalline regions are characterized by the formation of a repeating network of inter- and intramolecular hydrogen bonds, while the extended sheets formed through an expansion of these hydrogen-bonding contacts are further stabilized by van der Waals interactions, often referred to as ‘hydrophobic’ stacking planes. On the other hand, amorphous regions are poorly ordered regions with less regular interaction between the polymeric chains [3,4,5]. The reported percentages of crystalline versus amorphous material, as well as the size of the crystallites, are directly related to the cellulose source and physicochemical post-treatments. It has been reported that bleached kraft pulp samples from eucalyptus, which have smaller crystallites (4.5 nm), have reported lower crystallinity (60%) compared to those from pine, which have 5.0 nm crystallites and reported 65% crystallinity [6].

The crystalline structure of cellulose determines its accessibility to chemicals and enzymes and can be modified through changes in the rotational conformation of the -OH group on C6, resulting in a rearrangement of the molecular orientation and the network of intralaminar hydrogen bonds, which lead to the formation of different polymorphs, called cellulose I, II, III, and IV [3,7,8]. Cellulose I is the native polymorph in plants, characterized by parallel-packed chains [9]. This polymorph can be converted to cellulose II using a process called mercerization, with NaOH concentrations of between 8 and 12% (*w*/*w*) [10]. Most significantly, this induces a change in adjacent chain orientations, from parallel to antiparallel. This transformation is practically irreversible under typical processing conditions and induces changes in the intermolecular hydrogen bonding and increased space between chains [3,11,12]. These structural changes make the cellulose II-containing samples less crystalline, more accessible, and more reactive to subsequent treatments than the starting cellulose I-containing sample [13]. However, as the fibers dry, the interfibrillar spaces and other pores collapse, and the reactivity of cellulose II decreases [14].

Both polymorphs, cellulose I and II, can be converted to cellulose III_I_ and III_II_, respectively, by amine-based treatments such as liquid ammonia or ethylenediamine [15]. Ammonia and primary amines can incorporate themselves into the crystallite, mainly through hydrogen bonding, leaving the cellulose in an activated state and eventually inducing reorganization [16,17,18]. Conversion from cellulose I to cellulose II and III has been used as a strategy to activate cellulose fibers for chemical and enzymatic treatments [17,19,20].

Hyperspectral imaging (HSI) has recently emerged as a powerful extension of near-infrared (NIR) spectroscopy for the analysis of complex solid materials, providing both spectral and spatially resolved information in a single measurement. In contrast to conventional NIR spectroscopy, which yields only bulk-averaged spectra, HSI acquires a full spectrum at each pixel, enabling chemical imaging of heterogeneous samples and direct visualization of spatial gradients in composition and structure. This capability is particularly attractive for lignocellulosic materials, where the composition and structure of cellulose strongly influence reactivity, mechanical performance, and processability [21,22]. HSI presents a potential advantage for cellulose polymorph discrimination compared with other techniques, as it is non-destructive, fast, requires minimal sample preparation, offers the potential for spatial mapping of heterogeneity, and is suitable for industrial process control. By contrast, techniques such as XRD are non-destructive but require specific sample preparation, are time-consuming, and require expert analysis [23]. These methods complement each other: HSI serves as an initial screening tool, while XRD provides confirmation and precise quantification.

Therefore, we propose hyperspectral imaging based on NIR spectra as a rapid technique for the discrimination of cellulose I, II, III_I_, and III_II_ polymorphs, using three plant species commonly used for cellulose production: bleached kraft pulp of eucalyptus and pine and cotton linter fibers. Complementary techniques, namely X-ray diffraction (XRD) and X-ray photoelectron spectroscopy (XPS), were then applied to confirm the polymorph conversions and explain the structural and chemical changes observed by HSI-NIR.

## 2. Experimental

### 2.1. Material

Three types of raw materials were used; two were donated by a local pulp and paper company (Celulosa Arauco y Constitución S.A., Santiago, Chile): 1. Bleached eucalyptus kraft pulp (E), consisting of 80% *Eucalyptus nitens* and 20% *E. globulus*; 2. Bleached pine kraft pulp (P), consisting of *Pinus radiata*. The third sample is commercial zig-zag absorbent cotton (C) fibers purchased from a local supermarket (Hipermercado Tottus S.A., Concepción, Chile). The physicochemical properties (intrinsic viscosity and hemicellulose content) of the cellulose samples used in this work are presented in the Supplementary Information (Table S1).

The chemicals used in this work were sodium hydroxide pellets (≥99%), ethylendiamine for synthesis (≥99%), sulfuric acid solution (95–97%), potassium dichromate (≥99.9%), ammonium iron (II) sulfate hexahydrate (≤100%), and cupriethylendiamine (CED) solution (1 mol/L), all purchased from Sigma-Aldrich, St. Louis, MO, USA. Nanopure water (NPW) was used for treatment and solutions preparation, obtained by a Barnstead D4741 nanopure deionization system (Barnstead/Thermolyne Corp., Dubuque, IA, USA).

### 2.2. Preparation of Cellulose Polymorphs

The alkali treatment with NaOH at 7% *w*/*v* was used to remove residual hemicellulose from the feedstock, coded as NaOH7. In addition, NaOH at 20% *w*/*v* (NaOH20) was used to convert cellulose I to II. The NaOH treatment experimental procedure was followed based on those used in previous works of the research group [6,24].

NaOH solutions of 7% and 20% (*w*/*v*) were prepared to treat pulp at 10% (*w*/*v*) consistency. The treatment was carried out in polyethylene bags immersed in a water bath at 30 °C for 1 h. At the end of the treatment, the pulp was filtered and washed with plenty of water until a pH of 6 was reached, then the excess water was wrung out until the consistency was close to 30% (*w*/*v*).

Cellulose I was converted to cellulose III_I_ and cellulose II to III_II_ as follows. The fibers obtained from NaOH7 and NaOH20 were immersed in an ethylenediamine solution (≥99%) reaching a 10% (*w*/*v*) consistency and then allowed to stand for 24 h at room temperature. The obtained slurry was filtered and washed with anhydrous methanol until the pulp was free of the ammonia-like odor [25].

All cellulose polymorphs obtained from the different species are coded as shown in Table 1.

### 2.3. Hyperspectral Imaging

The hyperspectral camera Pika IR+ (Resonon Inc., Bozeman, MT, USA), operable in the range from 900 to 1700 nm, with 336 spectral channels, spectral resolution of 5.6 nm, and reflectance mode, was used to obtain an image of each sample in the form of a 0.01 g pellet (1 cm in diameter approximately) shaped in a hydraulic press to obtain spectral information as data cubes (three-dimensional spectral data cubes with two spatial and one spectral dimension) using contrast (class Stretch) as a filter tool and RGB (class TriBand) as a rendering tool. The analysis was performed using the software Spectronon Pro version 3.5.8 (Resonon Inc., Bozeman, MT, USA), where the hyperspectral cubes were obtained by selecting the entire surface of each sample. Spectra were subjected to discriminant analysis, with models trained on selected pixels from each cellulose polymorph. PeakFit software version 4.12 (Systat Software Inc., San Jose, CA, USA) was used for curve fitting to identify individual Gaussian peaks. Spectral normalization, differential analysis, and principal component analysis were performed using OriginLab software version 9.0.0 (Origin Lab Corporation, Northampton, MA, USA).

### 2.4. X-Ray Diffraction and X-Ray Photoelectron Spectroscopy

The effect of treatments on the crystalline cellulose was studied using XRD. Samples were prepared by pressing 50 mg of freeze-dried samples in a hydraulic press to form pellets. The pellets were placed in a sample holder, and X-ray diffractograms were recorded after mounting the sample holder on a D4 Endeavor X-ray diffractometer (Bruker AXS GmbH, Karlsruhe, Germany) using monochromatic Cu Kα radiation (λ = 0.154 nm) at 40 kV and 20 mA. Intensities were measured in the range 5° < 2θ < 45° with scan steps of 0.02°. Curve fitting was performed using PeakFit software version 4.12 (Systat Software Inc., San Jose, CA, USA) to identify individual peaks. In all cases, the F-number was >45,000 (r2 > 0.99). The crystallinity index (CrI) of the samples was calculated through peak-fitting, according to Equation (1) [23,26]:

(1) $$CrI\;(\%)=\frac{Acryst}{ATotal}\times 100$$

where Acryst is the sum of crystalline band areas, and ATotal is the total area under the diffractogram.

From the sum of peak area of the same crystal system (ΣACI for cellulose I, ΣACII for cellulose II, ΣACIII_I_ and ΣACIII_II_), cellulose I, II, III_I_ and III_II_ percentages were calculated using Equations (2), (3), (4), and (5), respectively [26]

(2) $$Cellulose\;I\;(\%)=\frac{\sum A_{CI}\;}{\sum A_{ACI+ACII}}\times CrI$$

(3) $$Cellulose\;II\;(\%)=\frac{\sum A_{CII}\;}{\sum A_{ACI+ACII}}\times CrI$$

(4) $$Cellulose\;{III}_{I}\;(\%)=\frac{\sum A_{C{III}_{I}}\;}{\sum A_{ACI+AC{III}_{I}}}\times CrI$$

(5) $$Cellulose\;{III}_{II}\;(\%)=\frac{\sum A_{C{III}_{II}}\;}{\sum A_{ACII+AC{III}_{II}}}\times CrI$$

The lateral crystallite size (L) was calculated from the Scherrer Equation (6) [27]

(6) $$L=\frac{k\;\times \;\lambda \;}{\beta \times \cos\theta }$$

where L is the size of the crystallite (nm), k is the Scherrer constant (0.96), λ is the X-ray wavelength, β is the full-width half-maximum (FWHM) of the (200) in cellulose I, (020) in cellulose II, (100/012/1–10) in cellulose III_I_ and cellulose III_II_ (100/1–10) reflection in radians, and θ is the Bragg angle of the respective characteristic reflection.

**Figure 1:**
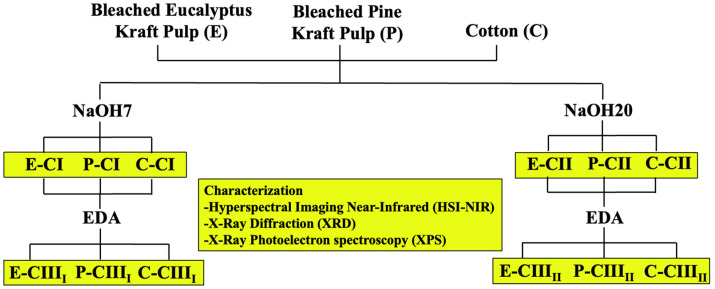
Schematic overview of the methodological workflow for cellulose polymorph preparation and characterization.

XPS analysis was performed on a FlexPS surface chemical analysis spectrometer equipped with an XR 50 (Al/Ag) dual-anode X-ray source, a FOCUS 500 monochromator, and a Phoibos 150 hemispherical analyzer (SPECS, Berlin, Germany). The samples were analyzed using an aluminum anode with Kα radiation at 1486.71 eV. The deconvolution of the 1s carbon peak into Gaussian components was performed in the PeakFit software version 4.12 (Systat Software Inc., San Jose, CA, USA). Figure 1 shows a schematic overview of the methodological workflow for cellulose polymorph preparation and characterization.

## 3. Results and Discussion

### 3.1. NIR Hyperspectral Imaging

**Figure 2:**
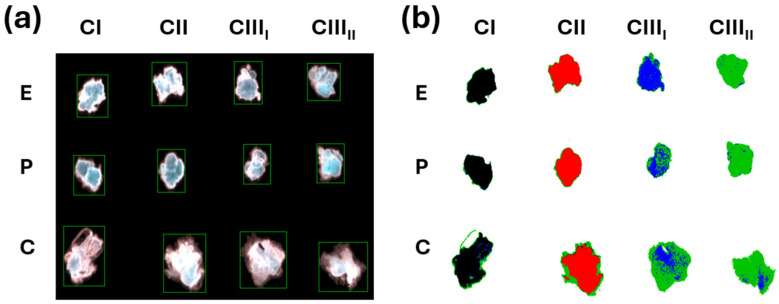
RGB images (**a**) without background and (**b**) subjected to linear discriminant classification, colormap.

To determine whether NIR hyperspectral imaging could discriminate between polymorphs, pellets made from cellulose I, II, III_I_, and III_II_ of eucalyptus, pine, and cotton fibers were placed in the NIR hyperspectral camera to generate RGB images shown in Figure 2. The background was first removed to avoid interfering with the analysis (Figure 2a). Then, the eucalyptus polymorph samples were stored as cubes and used to create predictive models to identify the pine and cotton polymorphs through linear discriminant analysis. The proof-of-concept of the classification of the polymorph using three botanical species, based on NIR images, resulted in colormap classes shown in Figure 2b. The colormap demonstrates successful identification of polymorphs by showing cellulose I in black, cellulose II in red, and cellulose III in a mixture of blue and green, regardless of the species used.

**Figure 3:**
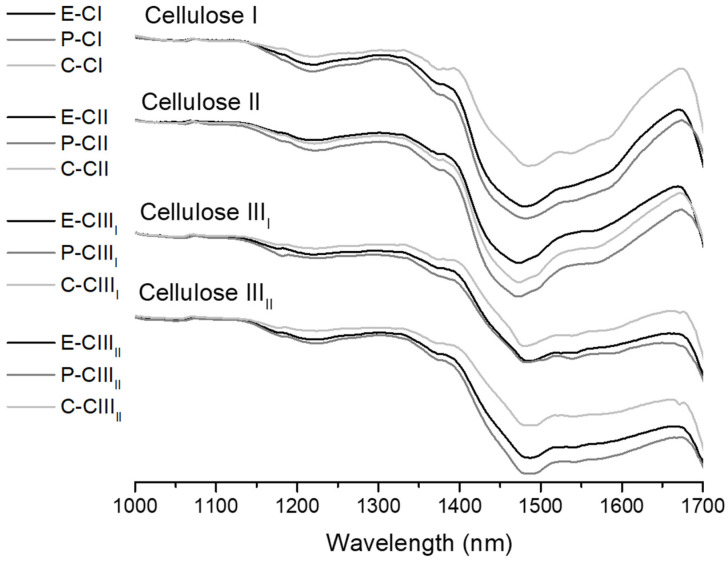
Spectral reflectance of cellulose I, II, III_I_, and III_II_ samples from eucalyptus, pine, and cotton.

The spectral reflectance obtained from the RGB images of the cellulose I, II, III_I_, and III_II_ samples, from eucalyptus, pine, and cotton, is shown in Figure 3. As in the linear discriminant classification colormap, the samples present different structures between the polymorphs observed from 1460 nm to 1700 nm.

**Figure 4:**
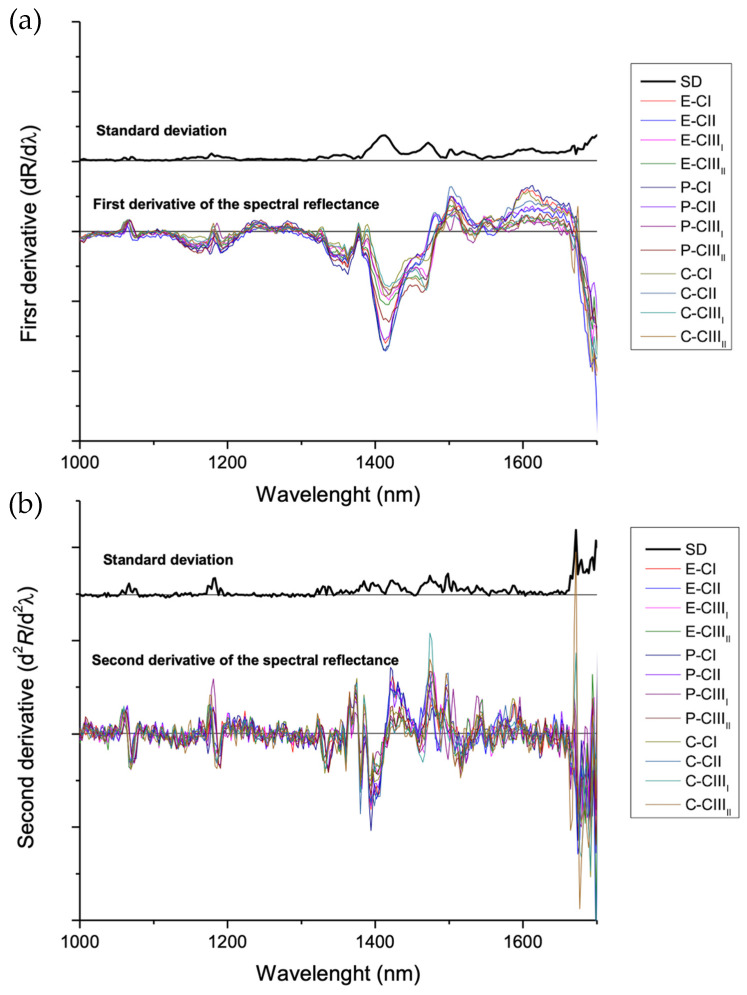
Differential analysis of (**a**) the first and (**b**) the second derivatives of the NIR spectra of cellulose I, II, III_I_ and III_II_ polymorphs from eucalyptus, pine, and cotton.

Initially, to identify the most important regions to analyze for distinguishing the cellulose polymorphs and separating them from the noise regions, differential analysis of the first (Figure 4a) and second derivatives (Figure 4b) of the NIR spectra was generated. The significant differences between the polymorph samples are expressed as peaks with higher or lower intensity in the 1375–1600 nm region. Specifically, the 1375–1480 nm region is mainly associated with the first overtones and combination vibrations of C–H stretching in cellulose and O-H stretching due to water adsorption [28]. The wavelength interval from 1480 to 1600 nm likely reflects shifts in the local hydrogen-bonding network, indicating differences in the supramolecular arrangement of the polymorphic forms of cellulose [29]. These last-mentioned regions were selected for principal component analysis (PCA) to emphasize the chemically relevant information while minimizing the influence of spectrally less informative or noisier regions.

**Figure 5:**
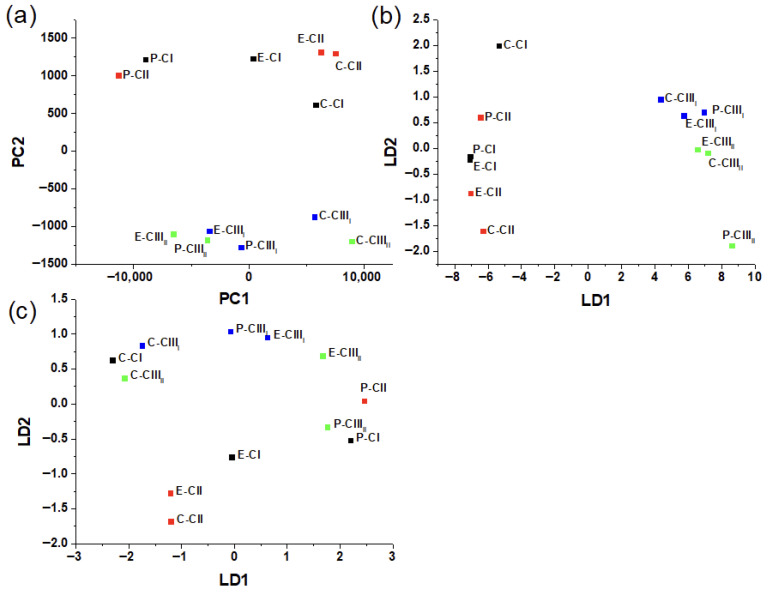
(**a**) PCA, (**b**) PCA-DA polymorphs, and (**c**) PCA-DA species.

The PCA performed on the 1480–1600 nm spectral interval is shown in Figure 5a. The cellulose samples form well-defined clusters that are clearly separated along PC1 and PC2, indicating that this wavelength range captures systematic spectral variance related to differences in hydrogen-bonding networks and crystalline packing, confirming that the structural changes between polymorphs are most prominently expressed in this region. The graph revealed two principal groups, one where cellulose I and II are relatively close to each other, and the other group where cellulose III_I_ and III_II_ are together. This behavior can be explained as cellulose I and II, despite their different chain packing, sharing similar types of intra- and inter-chain O–H hydrogen bonds. In contrast, cellulose III, produced by ammonia/amine treatment, presents a distinct unit cell and a substantially rearranged hydrogen-bonding network. When PCA is followed by discriminant analysis (PCA-DA) using the polymorph label (Figure 5b), the phenomenon of aggregation persists, but the spectral differences become clearer as cellulose III_I_ and III_II_ get more separated. Conversely, PCA-DA built with the botanical species (Figure 5c) as the class variable shows a more limited separation, indicating that in this spectral region, the variance associated with cellulose polymorphism is stronger and more structured than the differences linked to species.

**Figure 6:**
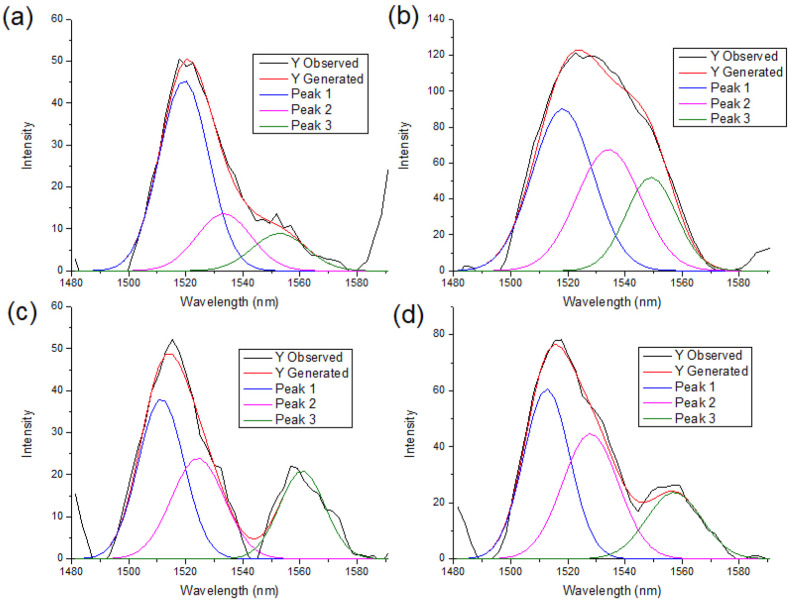
Deconvolution of peaks of eucalyptus samples in the 1480 to 1600 nm region. (**a**) E-CI; (**b**) E-CII; (**c**) E-CIII_I_; (**d**) E-CIII_II_.

**Figure 7:**
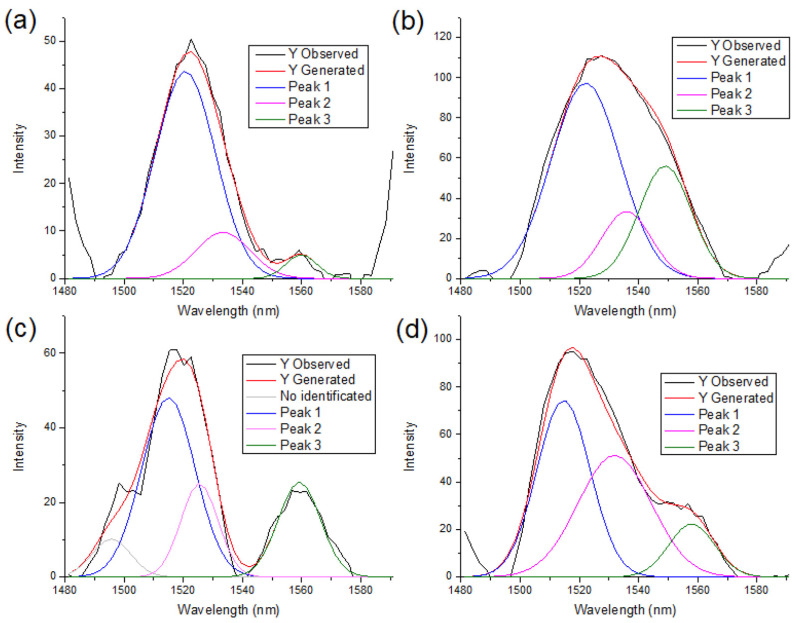
Deconvolution of peaks of pine samples in the 1480 to 1600 nm region. (**a**) P-CI; (**b**) P-CII; (**c**) P-CIII_I_; (**d**) P-CIII_II_.

**Figure 8:**
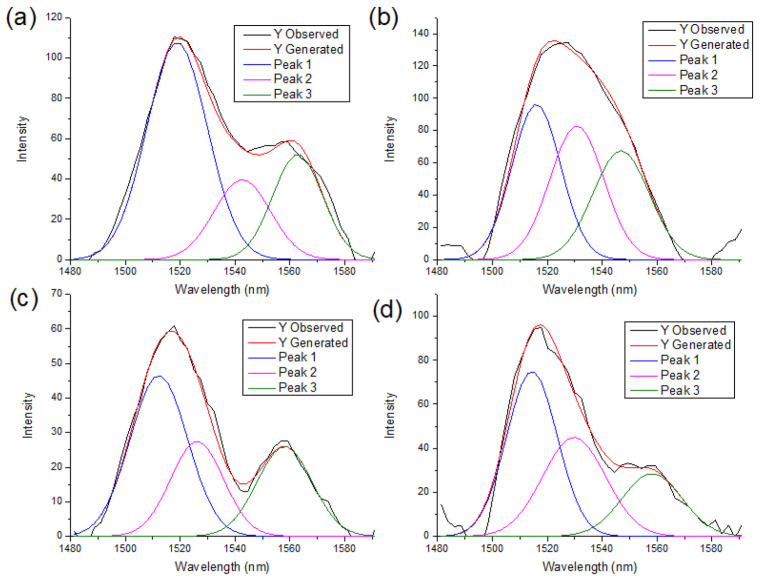
Deconvolution of peaks of cotton samples in the 1480 to 1600 nm region. (**a**) C-CI; (**b**) C-CII; (**c**) C-CIII_I_; (**d**) C-CIII_II_.

A deconvolution of the NIR spectra between the 1480–1600 nm region is presented in Figure 6, Figure 7 and Figure 8. For all polymorph samples, three principal peaks are identified, showing a consistent pattern across the three species, with the P-CIII_I_ (Figure 7c) sample being the only exception, presenting a peak at 1495 nm that does not fit in the polymorph structure.

The peak area percentage in Table 2 proves the dominance of Cellulose I by Peak 1, whereas conversion to cellulose II and III progressively decreases the relative intensity of Peak 1 and increases the contributions of Peaks 2 and 3, indicating a redistribution of hydroxyl environments associated with polymorphic transformation.

### 3.2. X-Ray Diffraction

**Figure 9:**
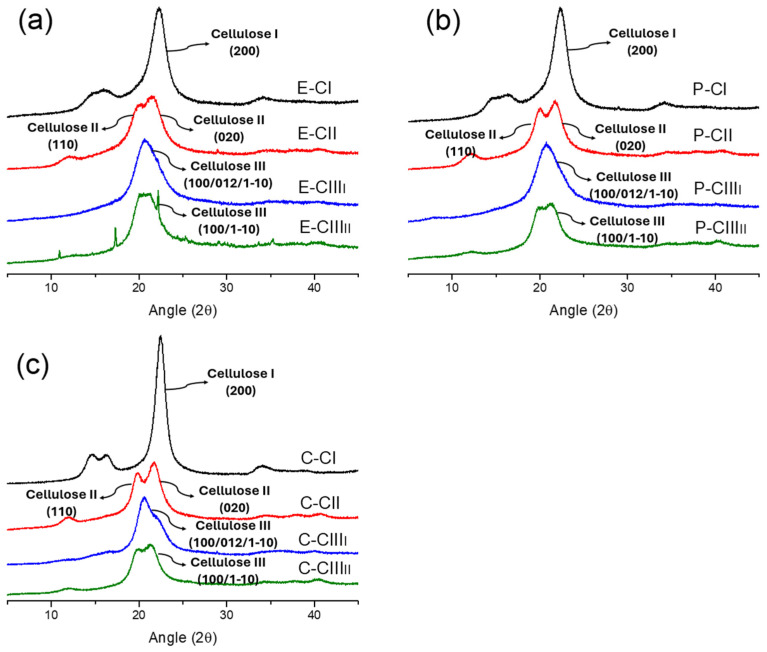
XRD diffractograms of cellulose I, II, III_I_, and III_II_ polymorphs from (**a**) eucalyptus, (**b**) pine, and (**c**) cotton.

XRD is a traditional technique to confirm the presence of cellulose polymorphs. In this case, the samples from each polymorph used in the hyperspectral imaging analysis were used to confirm the XRD patterns of cellulose I, II, III_I_, and III_II_ (Figure 9). Cellulose I was found at 14.8°, 16.5°, and 22.3° 2θ reflections assigned to the Miller indices (1–10), (110), and (200), respectively. The 18.5° 2θ reflection is assigned to the amorphous phase, and the 34.5° 2θ reflection is assigned to the (004) plane [26,30]. The NaOH treatment was successful in producing the cellulose II arrangement in all cases, displaying at 12.2°, 20°, and 22° the Miller indices (1–10), (110), and (020) of the reflecting planes [6,30]. The EDA treatment caused the conversion of cellulose I to III_I_ and cellulose II to III_II_. In the EDA-treated samples, the main visible peak is at 20.8°, corresponding to the (100/012/1–10) Miller index cluster in cellulose III_I_ and (100/1–10) Miller index in cellulose III_II_ [30,31]. The cotton sample also contained a residual peak contribution attributed to cellulose I (200) Miller index, indicating incomplete conversion from cellulose I. The incomplete conversion is also reflected in the RGB images subjected to linear discriminant analysis, showing a mixture of colors, especially in the samples of cotton cellulose III appearing more diffuse (Figure 2b).

Qin et al. [32] also showed a lack of complete conversion of cellulose I to III_I_ in corn stover samples soaked in 0.2–1 mL of EDA in a dry oven at 40–180 °C for a total of 40 min. The authors found that the treatment temperature and EDA loading influenced cellulose conversion. Higher loading decreased cellulose I content, while higher temperature from 20 °C to 100 °C decreased cellulose I content and resulted in higher cellulose III_I_ contents, i.e., less amorphous content. Increasing the temperature above 100 °C may lead to reorganization of the cellulose lattice, causing a reconversion to cellulose I. Taking this into account, the cotton pulp fiber morphology and higher molecular weight may make it less accessible to treatment with EDA. Therefore, to achieve a complete conversion of the cotton samples, it would be advisable to use EDA treatment at a higher temperature, without exceeding 100 °C. However, the potential effect on molecular weight reduction should also be considered.

**Figure 10:**
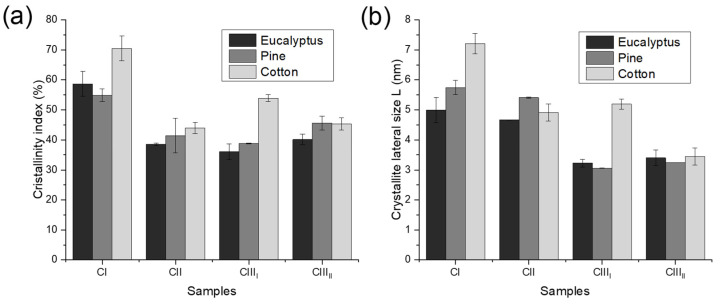
(**a**) CrI and (**b**) L of cellulose I (CI), cellulose II (CII), cellulose III_I_ (CIII_I_) and cellulose III_II_ (CIII_II_) from eucalyptus, pine, and cotton pulp.

The crystallinity index (CrI) values after treatment (Figure 10a) clearly show similar behavior regardless of species. The conversion of cellulose I, to either cellulose II or III_I_, resulted in a similar decrease in crystallinity between species. However, when cellulose II was converted to cellulose IIIII, the crystallinity remained unchanged, suggesting that prior strong alkaline treatment renders the crystalline regions more resistant to further reduction.

Figure 10b shows the change in lateral crystallite size (L) depending on the cellulose polymorph. All samples demonstrate decreases in L upon treatment. Undoubtedly, the most interesting change was seen in the cotton sample, which has the largest native C-CI L size (7.2 nm). This markedly decreased upon conversion to C-CII (4.9 nm), reaching the same size as those of the eucalyptus and pine samples, which had lower native L values. This may be related to the high purity of the cotton. The absence of hemicellulose may allow the sodium hydroxide to disrupt and break the cellulose chains more effectively, making them more amorphous. In the C-CI to C-CIII_I_ conversion, the cotton sample could not repeat the same phenomenon, which, although it resulted in a decrease, was not enough to reach the L of the other samples. In the case of the C-CIII_II_ samples, although the conversion from cellulose II to cellulose III_II_ does not decrease its crystallinity, it does reduce their crystallite lateral size, apparently by decomposition processes. All the species achieved a similar L, ranging from 3.246 to 3.4 nm.

### 3.3. X-Ray Photoelectron Spectroscopy

**Figure 11:**
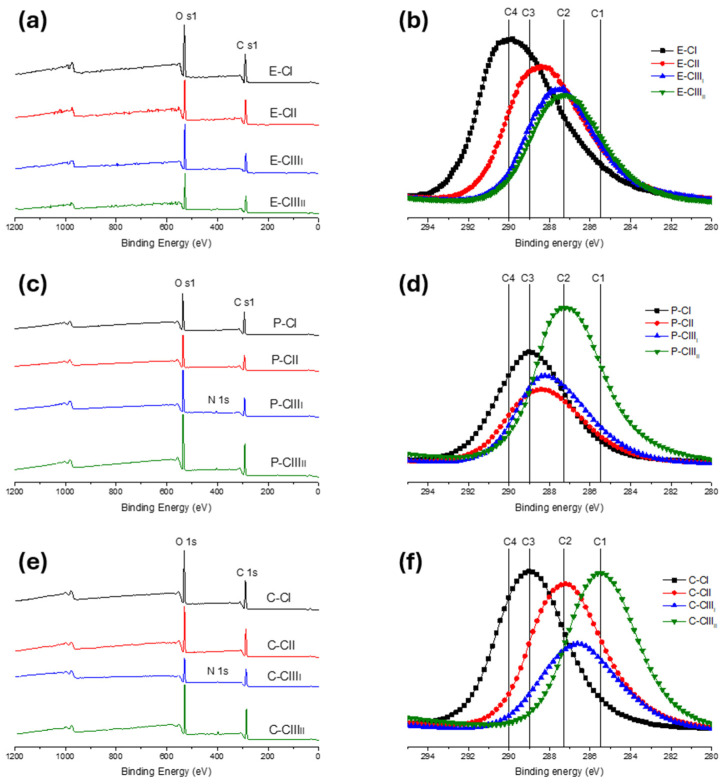
XPS spectra and C 1s identification peaks of cellulose I, II, III_I_, and III_II_ from (**a**,**b**) eucalyptus, (**c**,**d**) pine, and (**e**,**f**) cotton.

The differences in the concentration of elements present on the surface of the pulps were investigated using XPS. Figure 11 presents the XPS spectra of eucalyptus (Figure 11a), pine (Figure 11c), and cotton (Figure 11e) polymorph, showing the O 1s and C 1s peak characteristic of pure cellulose. In ethylenediamine-treated pine and cotton samples, a new peak appears at 400 eV, corresponding to nitrogen (N 1s) attributed to residual EDA [33]. The deconvoluted C1s XPS signals of each sample are shown in Figure 11b,d,f. The spectra were fitted in 285.5 eV, 287.3 eV, 289 eV, and 290 eV attributed to C1 (C-C), C2 (C-O), C3 (C=O), and C4 (COOH) [34].

The surface composition resulting from the deconvolution of C 1s is shown in Table 3. The C1 peak, corresponding to C-H/C-C, was observed to increase for all conversions: CI to CII conversion showed an increase of 11% in eucalyptus, 4.5% in pine, and 15% in cotton; CI to CIII_I_ conversion showed an increase of 19.7%, 9.2%, and 22.5% in eucalyptus, pine, and cotton; and the CII to CIII_II_ conversion showed an increase of 11.1%, 8.2%, and 68.5% for eucalyptus, pine and cotton. The same increasing trend was observed for C2 (C-O) in eucalypts, pine, and cotton samples in the conversion from CI to CII, changing from 14.6% to 30% in eucalypt samples, 21.3% to 31.1% in pine, and, in a higher way, cotton samples changed from 20% to 80.2%. The CI to CIII_I_ and CII to CIII_II_ of eucalyptus increased by 37.2% and 23.6%, and in pine by 17.6% and 51%. While cotton samples presented a different behavior in CIII, in CI to CIII_I_ increased by 60.2%, but in CII to CIII_II_ conversion, the content decreased from 72% to 11.7%.

C3 content (C=O; carbonyl) increased slightly by 4.7% in pine and eucalyptus during the conversion of CI to CII but decreased or became undetectable in the conversion to CIII, consistent with structural reorganization of cellulose. This result clarifies the differences in the exposure of the elements in the surfaces of the polymorph fibers, considering that the absence of C3 means that the element is not exposed.

As noted earlier, cellulose III presented an absence of C4 corresponding to COOH, while cellulose I showed the highest content, with pine and cotton showing 27.7% and eucalyptus pulp showing 44.3%. The COOH content in cellulose I is mainly explained by the different chemical processes, such as pulping and bleaching, which can lead to the oxidation of cellulose hydroxyl groups to carbonyls and then to carboxyl groups, especially in alkaline conditions [35,36,37]. Eucalyptus showed a higher COOH content due to residual xylan after NaOH7, confirmed by S18 (Table S1). When the alkali concentration was increased to 20% (NaOH20), the C4 decreased by 31.3%, due to xylan extraction, as their carboxyl groups make the molecule more soluble in sodium hydroxide solutions [38,39]. On the other hand, since cotton linters should be naturally free of COOH groups, the presented content would only be explained by the oxidation of the cellulose by the NaOH7 treatment; this effect was not observed with a higher NaOH20. In cellulose III, the increase in C1 and C2 content and the decrease in C4 are explained by the induction of EDA groups, which replace the COOH present in the cellulose and introduce more C-C bonds [40,41].

The HSI-NIR, XRD, and XPS results were compiled and analyzed using Pearson correlation analysis (Table 4). Peak 1 showed a moderate positive correlation with CrI and L, while being negatively correlated with the HSI-NIR deconvoluted Peak 3, indicating that a higher contribution of Peak 1 is associated with more ordered cellulose structures. In contrast, Peak 2 is positively correlated with the XPS C2% and strongly negatively correlated with the C3% component, which are assigned to carbon atoms bound to oxygen, suggesting that these NIR bands are sensitive to changes in surface hydroxyl and ether/acetals groups rather than to crystallinity. Overall, these results demonstrate that Peak 1 tracks crystallinity as determined by XRD, whereas Peaks 2–3 are more closely linked to the surface chemical state revealed by XPS. In summary, HSI-NIR provides a significantly faster alternative for accurate cellulose polymorph identification, requiring only 5–10 min per sample for spectral acquisition and discriminant analysis, in contrast to the hours needed for combined XRD (1–2 h) and XPS (30–60 min), not counting sample preparation and peak analysis time. While HSI-NIR is effective across polymorphs regardless of botanical origin, its sensitivity to detect subtle differences between cellulose types from different sources remains lower than that of XRD or XPS.

## 4. Conclusions

In this work, we demonstrated that near-infrared hyperspectral imaging, combined with chemometric analysis, enables rapid and non-destructive discrimination of cellulose I, II, III_I_, and III_II_ polymorphs in fibers derived from eucalyptus and pine kraft pulps and cotton linters. The 1480–1600 nm region of the NIR-HSI spectra provided a spectral fingerprint capable of separating polymorphs independently of botanical origin, consistent with the hydrogen-bonding rearrangements confirmed by XRD and XPS. Specifically, XRD was used as a traditional technique to confirm the expected polymorphic conversions, as well as to identify changes in crystallinity index and crystallite size upon transformation from cellulose I to II and III, while XPS indicated increased surface C-O functionality and loss of carboxyl groups in cellulose III, evidencing enhanced hydroxyl accessibility. Together, these results establish NIR-HSI as a powerful screening tool that can complement conventional diffraction and surface analysis techniques, offering a practical route to monitor cellulose polymorphism and associated changes in structure and reactivity in future cellulose-based material applications.

## Figures and Tables

**Table 1:** Treatment and labeling of cellulose I, II, III_I_, and III_II_ from fiber of eucalyptus, pine, and cotton.

.	Treatments	Sample Label
Eucalyptus Cellulose I	NaOH7	E-CI
Eucalyptus Cellulose II	NaOH20	E-CII
Eucalyptus Cellulose III_I_	NaOH7 followed by EDA	E-CIII_I_
Eucalyptus Cellulose III_II_	NaOH20 followed by EDA	E-CIII_II_
Pine Cellulose I	NaOH7	P-CI
Pine Cellulose II	NaOH20	P-CII
Pine Cellulose III_I_	NaOH7 followed by EDA	P-CIII_I_
Pine Cellulose III_II_	NaOH20 followed by EDA	P-CIII_II_
Cotton Cellulose I	NaOH7	C-CI
Cotton Cellulose II	NaOH20	C-CII
Cotton Cellulose III_I_	NaOH7 followed by EDA	C-CIII_I_
Cotton Cellulose III_II_	NaOH20 followed by EDA	C-CIII_II_

**Table 2:** Peaks area percentage (%) from deconvolution of 1480 to 1600 nm region.

Sample Label	Peak 1 (%)	Peak 2 (%)	Peak 3 (%)
E-CI	64.74	21.33	13.93
E-CII	44.07	34.89	21.04
E-CIII_I_	44.07	31.97	23.96
E-CIII_II_	42.42	37.83	19.75
P-CI	79.4	15.92	4.68
P-CII	59.58	14.6	25.82
P-CIII_I_	56.23	20.34	23.43
P-CIII_II_	45.43	42.91	11.66
C-CI	57.57	19.56	22.87
C-CII	36.24	34.43	29.33
C-CIII_I_	48.18	26.11	25.71
C-CIII_II_	45.67	33.94	20.39

**Table 3:** XPS analysis of cellulose I, II, III_I_, and III_II_ from eucalyptus.

Sample Label	C1 (C-H/C-C) (%) 285 eV	C2 (C-O) (%) 287 eV	C3 (C=O) (%) 289 eV	C4 (COOH) (%) 290 eV
E-CI	6.4	14.6	34.7	44.3
E-CII	17.4	30	39.6	13
E-CIII_I_	26.1	51.8	22.1	-
E-CIII_II_	28.5	53.5	18	-
P-CI	5.2	21.3	45.8	27.7
P-CII	9.7	31.1	50.5	8.7
P-CIII_I_	14.4	38.9	46.7	-
P-CIII_II_	17.9	82.1	-	-
C-CI	4.8	20	47.5	27.7
C-CII	19.8	80.2	-	-
C-CIII_I_	27.3	72.7	-	-
C-CIII_II_	88.3	11.7	-	-

**Table 4:** Pearson correlations between HSI-NIR, XRD, and XPS results.

		HSI-NIR			XRD		XPS			
		Peak 1	Peak 2	Peak 3	CrI	L	C1%	C2%	C3%	C4%
Peak 1	Pearson C.	1	−0.81603	−0.62839	0.50278	0.48006	−0.444	−0.60233	0.60975	0.471
	Sig.	-	0.00121	0.02864	0.09571	0.11421	0.14819	0.03821	0.10849	0.42328
Peak 2	Pearson C.	−0.81603	1	0.06314	−0.44257	−0.60931	0.44665	0.56051	−0.82117	−0.15943
	Sig.	0.00121	-	0.84543	0.14965	0.03545	0.14549	0.05801	0.01245	0.79787
Peak 3	Pearson C.	−0.62839	0.06314	1	−0.27248	−0.00886	0.1655	0.28565	−0.04255	−0.5577
	Sig.	0.02864	0.84543	-	0.39154	0.97819	0.60723	0.36811	0.92032	0.32867
CrI	Pearson C.	0.50278	−0.44257	−0.27248	1	0.78089	−0.27729	−0.31659	0.37676	0.70946
	Sig.	0.09571	0.14965	0.39154	-	0.00272	0.38291	0.31606	0.35758	0.17957
L	Pearson C.	0.48006	−0.60931	−0.00886	0.78089	1	−0.47316	−0.31505	0.60693	0.13246
	Sig.	0.11421	0.03545	0.97819	0.00272	-	0.12027	0.31854	0.11059	0.83184
C1%	Pearson C.	−0.444	0.44665	0.1655	−0.27729	−0.47316	1	−0.0978	−0.82504	−0.62776
	Sig.	0.14819	0.14549	0.60723	0.38291	0.12027	-	0.76236	0.01169	0.25686
C2%	Pearson C.	−0.60233	0.56051	0.28565	−0.31659	−0.31505	−0.0978	1	−0.67753	−0.98633
	Sig.	0.03821	0.05801	0.36811	0.31606	0.31854	0.76236	-	0.06487	0.00191
C3%	Pearson C.	0.60975	−0.82117	−0.04255	0.37676	0.60693	−0.82504	−0.67753	1	−0.61087
	Sig.	0.10849	0.01245	0.92032	0.35758	0.11059	0.01169	0.06487	-	0.27374
C4%	Pearson C.	0.471	−0.15943	−0.5577	0.70946	0.13246	−0.62776	−0.98633	−0.61087	1
	Sig.	0.42328	0.79787	0.32867	0.17957	0.83184	0.25686	0.00191	0.27374	-

## Data Availability

The original contributions presented in this study are included in the article. Further inquiries can be directed to the corresponding author.

## Supplementary Materials

The following supporting information can be downloaded at: https://www.mdpi.com/article/10.3390/polym18091047/s1.
