# Supplementary material for: Discrimination of Cellulose I, II, IIII and IIIII Polymorphs in Cellulosic Fibers by NIR Hyperspectral Imaging Supported by XRD and XPS
Source: Polymers (Basel). 2026 Apr 25;18(9):1047. doi: 10.3390/polym18091047 (PMC13164972; doi:10.3390/polym18091047)
Supplement: Supplementary file 1 [file polymers-18-01047-s001.zip › polymers-4252089-supplementary.pdf]

Supplementary information

Metodology

S18. Polymorphs obtained from NaOH treatments were characterized using the S18 alkaline solubility method (TAPPI T235 cm-00 standard) to determine the percentage of low molecular weight carbohydrates (hemicellulose and degraded cellulose). This method is based on treating the pulp with an 18% NaOH solution at 25 °C for 1 hour. The pulp is then filtered, and the filtrate is oxidized with potassium dichromate and titrated with ferrous ammonium sulfate.

Intrinsic viscosity. The intrinsic viscosity of the pulp (TAPPI T230 om-99 standard) was measured using the capillary viscometer method.

Results

The cellulose polymorphs were characterized using the S18 method. The amount of residual hemicelluloses in the cellulose polymorph samples was estimated with the S18 % in the fibers, to assess the efficacy of NaOH. The analysis of S18 in Table 2 shows that the NaOH treatment effectively extracted residual hemicelluloses from the BEKP and BPKP pulps, while the native absence of hemicelluloses in the cotton fibers confirmed the effectiveness of the analysis, resulting in a S18 close to 0%.

In general, the results show that chemical treatments caused a reduction in cellulose chain length. In NaOH-treated samples, the reduction effect was small when NaOH20 was compared to NaOH7, changing the intrinsic viscosity from 710 ml/g to 632 ml/g from E-CI to E-CII samples. For P-CI to P-CII samples, the decrease was from 531 ml/g to 508 ml/g.

Cotton samples showed a more pronounced effect of NaOH20, with a reduction in intrinsic viscosity from 1003 ml/g (C-CI) to 677 ml/g (C-CII).

Table S1 Alkali solubility (S18) of cellulose I, II, III_I, and III_{II} from fiber of eucalyptus, pine, and cotton.

**Sample label**	**S18 (%)**	**Intrinsic viscosity (mL/g)**
E-CI	2.36 ± 0.04	710 ± 2
E-CII	1.11 ± 0.05	632 ± 8
E-$CIII_I$	-	107 ± 2
E-$CIII_{II}$	-	94.4 ± 0.4
P-CI	5.2 ± 0.3	531 ± 11
P-CII	1.42 ± 0.05	508 ± 14
P-$CIII_I$	-	107 ± 10
P-$CIII_{II}$	-	143 ± 3
C-CI	0.73 ± 0.04	1003 ± 3
C-CII	0.27 ± 0.07	677 ± 29
C-$CIII_I$	-	726 ± 50
C-$CIII_{II}$	-	154 ± 4
